# Improving diagnosis of acute pulmonary edema in pregnancy for saving lives through imaging analysis: Case report and literature review

**DOI:** 10.1097/MD.0000000000042517

**Published:** 2025-05-16

**Authors:** Chao-Xia Song, Yu Yang, Jin-Xia Wei, Pei Chi, Xiao-Juan Peng

**Affiliations:** a Department of Cardiology, Renmin Hospital of Qingxian, Cangzhou, China; b Department of General, General Hospital of Central Theater Command of the People’s Liberation Army, Wuhan, China; c Department of Obstetrics, Renmin Hospital of Qingxian, Cangzhou, China; d Department of Obstetrics and Gynaecology, Guangzhou Development District Hospital (Guangzhou Huangpu District People’s Hospital), Guangzhou, China.

**Keywords:** hypoxia, postpartum, pregnancy, pulmonary edema, radiographic imaging, ultrasonic diagnosis

## Abstract

**Rationale:**

Acute pulmonary edema is relatively uncommon during pregnancy. Due to its easy misdiagnosis or delayed diagnosis, the mortality rate of acute pulmonary edema in the postpartum period is very high. There is limited data in the literature on imaging analysis of acute pulmonary edema in the postpartum period, which may contribute to its misdiagnosis or delayed diagnosis.

**Patient concerns:**

This case describes a 28-year-old woman who was admitted to the respiratory intensive care unit with dyspnea that had been misdiagnosed as pneumonia 3 days after delivery.

**Diagnoses:**

After careful radiographic imaging analysis and bedside ultrasound monitoring, the patient was finally diagnosed with acute pulmonary edema.

**Interventions:**

Subsequently, oxygen inhalation, diuresis, human albumin supplementation, and other treatments were administered.

**Outcomes:**

Her symptoms basically disappeared within 1 day, and the patient did not report any discomfort with 7 months of follow-up.

**Lessons:**

It has highlighted the importance of analyzing radiographic findings in the diagnosis of acute pulmonary edema during the postpartum period in this case. This analysis of radiographic findings requires multidisciplinary consultation between obstetricians and other specialties in the management of maternal health. It also reflects that early diagnosis and timely intervention are key to improving postpartum acute pulmonary edema.

## 1. Introduction

Any factor that reduces blood colloid osmotic pressure, increases capillary permeability, or increases venous pressure can lead to extravasation of intravascular fluid and an increase in interstitial fluid volume.^[1,2]^ If this fluid accumulates abnormally in the interstitial and alveolar spaces, pulmonary edema is formed.^[3,4]^ Due to the physiological changes associated with pregnancy, pregnant women are more likely to develop acute pulmonary edema.^[5]^ After the occurrence of pulmonary edema, reduced lung compliance and oxygen exchange disorders, arterial hypoxia, acidosis, hypercapnia, and even respiratory failure. The development of pulmonary edema during pregnancy is associated with increased maternal and fetal morbidity and mortality.^[6]^ There are a lot of literature on the diagnosis of tumor lesions and pneumonic lesions by radiography,^[7,8]^ but very few literature on imaging analysis of acute pulmonary edema by radiography. In this report, we describe a delayed diagnosis case of acute pulmonary edema after delivery achieved a rapid recovery. We believe that favorable outcomes can be ensured by radiographic imaging analysis and bedside ultrasound monitoring, which increases awareness of postpartum acute pulmonary edema.

## 2. Case report

A 28-year-old woman was transferred to the respiratory intensive care department on the 3rd day after vaginal delivery, presenting with dyspnea, cough, and sputum. Sputum is frothy sputum with blood, and the amount of sputum in 2 hours is about 20 mL. The obstetrician gave the initial diagnosis of pneumonia.

The patient did not smoke and had no contact with pets or poultry during pregnancy. And she had intermittent lower limb edema in the 7th month of pregnancy, which was mainly in the evening and relieved after rest. At that time, she was only found thyroid stimulating hormone of 7.028 mIU/L (normal range: 0.35–5.1 mIU/L), free thyroxine of 8 pmol/L (normal range: 11.20–23.81 pmol/L) on blood tests, Significant positive results were not observed in laboratory findings on anemia indicators, trace elements, vitamins. Blood clotting function, liver and kidney function were also normal, and the laboratory tests are shown in Table 1. The patient’s blood pressure and respiratory function were also in normal. She was treated with 50 µg of levothyroxine sodium orally daily. Then, her blood pressure fluctuated between 140–110/80–70 mm Hg 1 month before delivery, and no other drugs were taken. The patient had a poor appetite during labor and was treated with intravenous fluids to establish venous access. Although she was a primipara, the labor was smooth, and no oxytocin was used. Her full-term boy baby, whom was born via natural vaginal delivery by right lateral perineal incision midwifery at 39 weeks and 5 days of gestation, weighing 3.3 kg. The baby Apgar score was 9 (A + P + G + A + *R* = 2 + 2 + 2 + 1 + 2) after 1 minute, and 10 (A + P + G + A + *R* = 2 + 2 + 2 + 2 + 2 + 2) after 5 minutes.

**Table 1:** Laboratory test results of the patient.

Laboratory tests	Normal range	Results at pregnancy	Results at postpartum
WBC (×10^9^/L)	4–10	5.19	13.05
RBC (×10^12^/L)	3.5–5.0	4.29	3.12
Hemoglobin (g/L)	110–150	124	108
PLT (×10^9^/L)	100–300	216	296
C-reactive protein (mg/L)	<22	6.22	84.02
Erythrocyte sedimentation rate (mm/h)	0–10	4.5	12.6
Procalcitonin (ng/mL)	<0.5	0.350	0.247
Interleukin-6 (pg/mL)	0–40	22.6	44.2
BNP (pg/mL)	0–100	98	82
Albumin(g/L)	35–55	36.6	32.2
Alanine aminotransferase (U/L)	7–40	28.2	18.1
Aspartate aminotransferase (U/L)	13–35	20.4	35.0
Direct bilirubin (µmol/L)	0–6.8	3.2	4.3
Creatinine (µmol/L)	41–81	48.7	44.8
Urea nitrogen (µmol/L)	3.6–9.5	4.19	6.21
Thyroid stimulating hormone (mIU/L)	0.35–5.1	7.028	4.412
Triiodothyronine (nmol/L)	0.89–2.49	1.56	1.47
Thyroxin (nmol/L)	64.36–186.64	82.39	66.12
Free thyroxine (pmol/L)	11.20–23.81	8.0	0.8
Free triiodothyronine (pmol/L)	2.76–6.45	3.43	2.95
Protein (urinalysis routine)	Negative	0	–
Urobilinogen (urinalysis routine)	Negative	0	–
Occult blood (urinalysis routine)	Negative	0	–

BNP = B-type natriuretic peptide, PLT = platelet, RBC = red blood cell, WBC = white blood cell.

**Figure 1.**
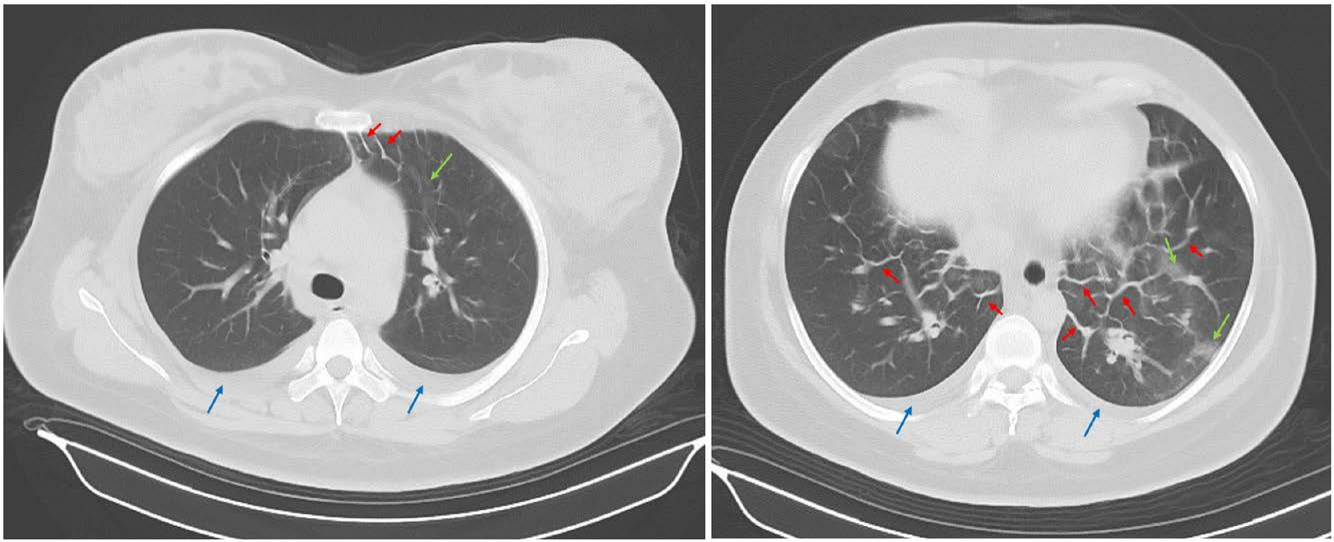
16-Slice computerized tomography of chest examination revealed small bilateral pleural effusions (blue arrows), interstitial inflammatory changes (green arrows), and bilateral pulmonary interlobular septal thickening (red arrows).

**Figure 2.**
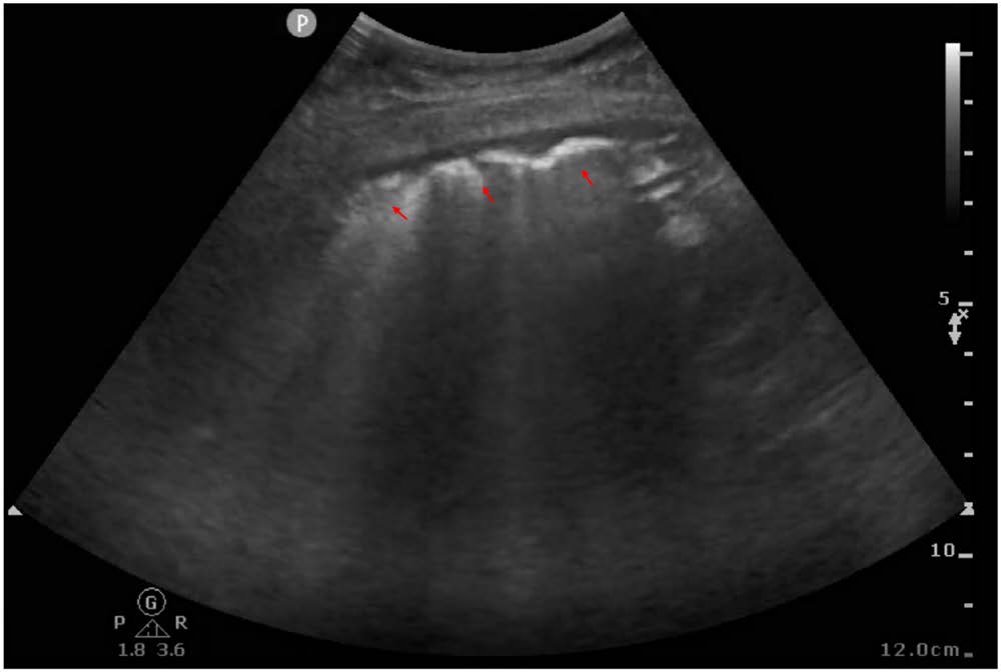
Bedside ultrasound image of chest examination revealed focal diffuse B-line chest wall with pleural slip (red arrows) on the left side.

**Figure 3.**
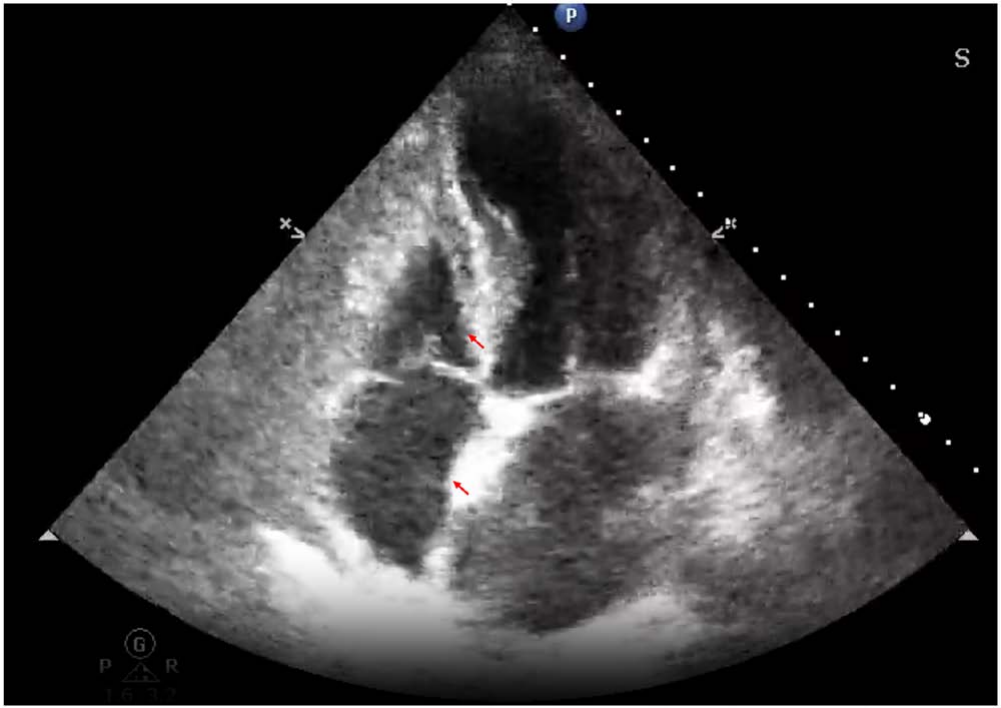
Bedside ultrasound image of transthoracic echocardiography showed that the inner diameter of the right atrium and right ventricle decreased significantly (red arrows).

On the time of transfer, she exhibited a body temperature of 37.5°C, a heart rate of 110 beats/min, blood pressure of 140/90 mm Hg, respiratory rate of 36 breaths/min, and oxygen saturation levels of 78% without supplemental oxygen and 90% with oxygen. Lips slightly purple, lungs scattered in moist rales, heart rhythm, cardiac auscultation did not hear, and murmurs. Mild edema of both lower limbs. Blood tests showed a white blood cell count of 13.05 × 10^9^/L, neutrophil count of 11.36 × 10^9^/L, neutrophil percentage of 86.2%, and the free thyroxine levels were lower. The comparative laboratory tests are shown in Table 1. The patient underwent a chest computed tomography examination, which revealed small bilateral pleural effusions (Fig. 1, blue arrows), interstitial inflammatory changes (green arrows), and bilateral pulmonary interlobular septal thickening (red arrows). After careful radiographic imaging analysis by multidisciplinary team consultation between obstetricians and other specialties, diagnoses of postpartum acute pulmonary edema were highly considered. In this discussion, experts suggested that patients could be considered for real-time monitoring with bedside ultrasound, including lung ultrasound (Fig. 2) and cardiac ultrasound (Fig. 3). Transthoracic pulmonary ultrasound revealed partial lung consolidation, a small amount of pleural effusion, and focal diffuse B-line chest wall with pleural slip, which was considered to have induced acute pulmonary edema. Transthoracic echocardiography showed that the inner diameter of the right atrium and right ventricle decreased significantly, and the displacement of the tricuspid valve ring during systolic and diastolic periods increased significantly (31.4 mm), indicating the state of hyperdynamic contraction of the right ventricle. At the same time, the left ventricular septal motion was enhanced, but the apex of the heart was almost immobile. Combined with the patient’s no special history, it was considered that the patient’s acute pulmonary edema induced cardiomyopathy.

Then, she received high-flow oxygen intake, human albumin supplementation (10 g, intravenously), and diuretic (furosemide, 5 mg, intravenously). Her symptoms basically disappeared within 1 day, and no follow-up imaging re-examine was conducted considering the will of pregnant women. After 7 months of follow-up, the patient did not report any discomfort. She expressed great satisfaction with the diagnosis and treatment of the medical visit, especially acute pulmonary edema.

## 3. Discussion

There are several potential causes of postpartum dyspnea, such as pulmonary embolisms, amniotic fluid embolisms, and peripartum cardiomyopathies (PPCM), but the acute pulmonary edema occurs less frequently.^[9]^ Only 0.08% of pregnant and postpartum women reported this disease.^[9]^ Although rare, it is a life-threatening disease with high maternal and perinatal mortality. More than half of these diagnosed cases were related to tocolytic therapy or cardiac disease, and the remainder to pre-eclampsia or iatrogenic volume overload.^[10]^ The use of oxytocin or calcium channel antagonists can lead to serious complications associated with this disease.^[11]^ Heart failure, pulmonary embolism, and sepsis are common misdiagnoses for this disease.^[12]^ In this case, there was no clear cause for the acute pulmonary edema that occurred in the postpartum. Iatrogenic volume overload needs to be considered as a cause disposition of acute pulmonary edema postpartum in this case.

The key to obtaining a correct diagnosis is recognizing that the patient has typical cough with bloodshot foam sputum, pregnancy-induced hypertension, hypoproteinemia, and a small pleural effusion. The patient’s body temperature was not significantly elevated, and the procalcitonin was normal. Moreover, acute respiratory failure caused by infection is less likely. The occurrence of pneumonia often originates in the lungs and has little relationship with cardiac function.^[13,14]^ Therefore, simple pneumonia is often unintentional dysfunction. Patients with pulmonary embolism or amniotic fluid embolism usually have chest pain, hemoptysis, dyspnea symptoms, and rarely have pleural effusion.^[15]^ Pregnancy-related heart failure mostly occurs in older women with underlying heart disease, and clinical symptoms should begin as early as 3 months after pregnancy.^[16,17]^ PPCM is a rare but serious condition that occurs in the last month of pregnancy or within 5 months after delivery. It is characterized by the development of heart failure due to left ventricular systolic dysfunction, which can lead to acute pulmonary edema, a life-threatening condition where fluid accumulates in the lungs, causing severe respiratory distress. Echocardiographic evaluation is crucial in diagnosing and managing PPCM, as it provides detailed information about cardiac structure and function. Echocardiography can reveal left ventricular dilation and reduced ejection fraction, which are hallmark features of PPCM.^[18]^ PPCM could be ruled out by bedside ultrasound images from transthoracic echocardiography, which showed that the inner diameters of the right atrium and right ventricle decreased significantly in this patient. Biochemical markers can also aid in the diagnosis and prognosis of PPCM. Elevated levels of natriuretic peptides, such as B-type natriuretic peptide (BNP) or N-terminal pro-BNP, are often observed in PPCM and correlate with the severity of heart failure.^[19,20]^ This patient’s BNP result was normal, which added to the difficulty of diagnosis.

The diagnosis of this case is challenging, but not without clues. On the one hand, there are the clinical manifestations of the previous analysis. On the other hand, there are educational computed tomography imaging analysis and bedside ultrasound monitoring findings. Bilateral pulmonary interlobular septal thickening, small bilateral pleural effusions, interstitial inflammatory changes, all these are helpful for diagnosis.^[6,12]^ In particular, the bilateral pulmonary interlobular septal thickening. Typically, only a single interlobular septal attachment to the pleura is observed in the anterior mediastinum, but multiple in this case. Interlobular septal thickening can be smooth, irregular (spiculated), or nodular.^[21–23]^ Smooth interlobular septal thickening, as seen in this instance, is commonly associated with various venous, lymphatic, and infiltrative pathologies, notably pulmonary edema.^[24]^ Acute pulmonary edema is supposed to be an exudative lesion in both lungs, but this patient had left lung lesions. After careful observation and analysis, we found that the patient always prefers the left side position after delivery. And what is causing her to be on her left side is pain from the vaginal incision when she is on her right side.

Acute pulmonary edema postpartum changes rapidly and critically, and even sudden death. Early and accurate diagnosis is the key to treatment. Immediate management of this disease includes oxygenation, circulation control with venodilators, supplement albumin, avoiding the use of intubation and mechanical ventilation. If it starts with left heart dysfunction, followed by acute pulmonary edema, then right heart dysfunction and inferior vena cava dilatation and fixation, this is a typical manifestation of volume overload. This is equivalent to the right cardiac discharge is higher than the left cardiac discharge, this blood volume is poor in the pulmonary circulation, resulting in acute pulmonary edema. The direction of treatment should consider diuresis, dehydration. Otherwise, if the right heart and the left heart do not match, the right heart exercise stress is enhanced, and the liquid that is not originally loaded is pushed into the lung, resulting in local volume overload (that is, acute pulmonary edema, no congestion in other systemic circulation organs), then the cardiotonic becomes the main direction.^[25,26]^

## 4. Limitations

This case report analyses the radiographic details of acute pulmonary edema and does not imply that all patients with acute pulmonary edema have these radiographic findings.

## 5. Conclusion

In conclusion, the diagnosis and treatment of postpartum acute pulmonary edema were challenged. Only emphasizing treatment-related clinical experience is of limited benefit to obstetricians. However, the management model that emphasizes imaging analysis to obtain early diagnosis and then accurate treatment is more worthy of recommendation.

## Author contributions

**Data curation:** Chao-Xia Song, Pei Chi, Xiao-Juan Peng.

**Methodology:** Chao-Xia Song, Yu Yang, Jin-Xia Wei, Pei Chi, Xiao-Juan Peng.

**Software:** Chao-Xia Song, Yu Yang, Jin-Xia Wei, Xiao-Juan Peng.

**Conceptualization:** Yu Yang, Jin-Xia Wei, Xiao-Juan Peng.

**Funding acquisition:** Yu Yang, Jin-Xia Wei, Xiao-Juan Peng.

**Investigation:** Yu Yang, Jin-Xia Wei.

**Project administration:** Yu Yang, Jin-Xia Wei, Xiao-Juan Peng.

**Supervision:** Yu Yang, Jin-Xia Wei.

**Writing – review & editing:** Yu Yang, Jin-Xia Wei, Xiao-Juan Peng.

**Formal analysis:** Jin-Xia Wei.

**Resources:** Jin-Xia Wei, Pei Chi.

**Validation:** Jin-Xia Wei.

**Visualization:** Jin-Xia Wei.

**Writing – original draft:** Jin-Xia Wei, Xiao-Juan Peng.
